# Hippocampal and Prefrontal Cortical Brain Tissue Levels of Irisin and GDF15 Receptor Subunits in Children

**DOI:** 10.1007/s12035-020-02250-4

**Published:** 2021-01-07

**Authors:** Travis C. Jackson, Kiersten Gorse, Jeremy R. Herrmann, Patrick M. Kochanek

**Affiliations:** 1 University of South Florida, Morsani College of Medicine, USF Health Heart Institute, MDD 0742, 560 Channelside Dr, Tampa, FL 33602 USA; 2 Department of Molecular Pharmacology & Physiology, University of South Florida, Morsani College of Medicine, 12901 Bruce B Downs BLVD, MDC 2532, Tampa, FL 33612-4799 USA; 3 School of Medicine, Children’s Hospital of Pittsburgh of UPMC, Safar Center for Resuscitation Research, University of Pittsburgh, John G. Rangos Research Center – 6th Floor, 4401 Penn Avenue, Pittsburgh, PA 15224 USA; 4 Department of Critical Care Medicine, School of Medicine, University of Pittsburgh, Scaife Hall 3550 Terrace Street, Pittsburgh, PA 15213 USA

**Keywords:** Integrin-αV, Integrin-β5, GFRAL, Hippocampus, Prefrontal cortex, Zika virus

## Abstract

Cold-stress hormones (CSHs) stimulate thermogenesis and have direct neuroprotective effects on the brain. The obligatory receptor components of two new CSHs (irisin and growth differentiation factor-15 [GDF15]) were recently discovered. Irisin binds integrin-αV/β5 heterodimers while GDF-15 binds to the orphan receptor glial cell-derived neurotrophic factor (GDNF) family receptor α-like (GFRAL). In addition, integrin-αV/β5 was just identified as the key receptor mediating Zika virus infection in the CNS. We measured integrin-αV, integrin-β5, and GFRAL protein levels across 78 high-quality human male/female brain tissues in infants, toddlers, preschoolers, adolescent, and adults—providing the most robust analysis to date on their levels in the human cortex and hippocampus. We report that integrin-αV was detected at all ages in the prefrontal cortex with levels greatest in adults. Integrin-αV was also detected in the hippocampus in all age groups. In contrast, integrin-β5 was detected in cortex and hippocampus largely restricted to infants. Co-expression of integrin-αV/β5 in the human infant hippocampus and cortex suggests the possibility that irisin has a more robust effect on the developing vs. the adult brain and may have implications for Zika virus infection in infants and young children.

**Supplementary Information:**

The online version contains supplementary material available at 10.1007/s12035-020-02250-4.

## Introduction

Cold-stress hormones (CSHs) are broadly defined as hormones that increase their production and/or secretion in response to cold-stressors [1]. They are part of an integrated network of cold-defense mechanisms that are recruited by the body to protect homeotherms from sudden decreases in ambient temperature [1]. CSHs activate thermogenic heat-producing signaling pathways to maintain normothermia—or slow the progression of hypothermia—but also exert a wide range of additional biological effects on numerous organs including the brain [1]. The list of CSHs continues to grow and includes well-known members like fibroblast growth factor 21 (FGF21), in addition to newer members like irisin and growth differentiation factor-15 (GDF15), among others.

Germane to FGF21, and in particular its key receptor binding target β-klotho, we previously reported that β-klotho levels were remarkably abundant in the hippocampus and cerebral cortex in human infants, low in toddlers, and near absent in adolescents and adults [2, 3]. Prior to these studies, β-klotho was not thought to be expressed in the hippocampus and cerebral cortex at any age [4]. Discovery that brain FGF21 signaling pathways may be more robust in infants has raised important clinical questions [5]. For instance, exogenous FGF21 therapy is robustly neuroprotective in rodent models of perinatal asphyxia but whether its benefits are mediated by direct activation of neuronal β-klotho receptors in the brain or by indirect mechanisms in the periphery (or both) remains to be elucidated [6]. Also, it is unclear to what extent standard of care 72 h therapeutic hypothermia (TH), used in term newborns with perinatal asphyxia, triggers FGF21 secretion. Studies to address these questions are underway and thus far suggest critical importance of defining CSH receptor levels in the human brain at different ages to understand the mechanisms potentially targeted by cerebroprotective cooling in a variety of populations [2, 3]. Those studies may also provide additional insights relevant to neurodevelopment and/or other disease processes in humans. Additional CSH ligands secreted during cold-stress may also affect neuronal survival in brain regions or exhibit other effects where their receptors are expressed.

Irisin is a CSH derived from the cleavage of the transmembrane protein fibronectin type III domain containing 5 (FNDC5) [7]. While PK/PD studies on its distribution and clearance from the brain are lacking, irisin is thought to cross the blood brain barrier based on its presence in human CSF, and on observations that gene expression in the brain is altered following peripheral administration in rodents [8]. The molecular weight of irisin varies (~ 13–25 kilodaltons/kDa) due to post-transcriptional glycosylation [9]. Exogenous irisin therapy is neuroprotective in rodent brain injury models—supporting the notion that it may have direct protective effects in the CNS when secreted into the systemic circulation [10, 11]. In humans, cold-induced shivering increases irisin secretion from skeletal muscle, hence its categorization as a CSH [12]. Also, Ayden and colleagues reported that serum irisin levels are increased during the TH-cooling phase of cardiopulmonary bypass in adult patients undergoing heart surgery, and levels decrease once returned to normothermia [13]. Recently, Kim and colleagues identified integrin-αV/β5 heterodimers as the primary receptor signaling complex that mediates intracellular responses to circulating irisin [14]. To better understand if age influences CNS responses to stimuli that increase irisin levels (such as cold-stress), studies are needed to define the protein levels of irisin receptor components in the developing vs. the mature human brain, including in injury-prone brain regions like the hippocampus [15].

Secondary to our interest in irisin receptors as it relates to hypothermia, but also of major clinical importance, integrin-αV/β5 heterodimers were discovered to mediate Zika virus infection in neural progenitor cells [16, 17]. Zika is an RNA flavivirus and a human pathogen [18]. Localized outbreaks have been recorded throughout Africa and Asia since its discovery in 1947. However, the 2015–2016 pandemic in the Americas raised alarm germane to its emerging global threat to public health, including in the continental US [18, 19]. Furthermore, increases in global temperature have expanded the geographical range of the *Aedes aegypti* mosquito, which is the main vector that mediates Zika transmission in humans [20]. Notably, unlike other pathogenic flaviviruses, Zika preferentially affects the nervous system and can produce a spectrum of neurological complications [18]. The most devastating sequalae are seen in newborns exposed in utero, which can result in microcephaly at birth, among other severe deficits in brain function [21]. The incidence of birth defects is 4–15% [18]. However, 9% of newborns negative for birth defects had at least one neurodevelopmental abnormality by 2 years of age [22]. At present, vaccines to prevent the complications of infection during pregnancy are under development but as of yet unavailable [18]. Moreover, and highly relevant to our neurodevelopmental characterization of integrin-αV/β5 protein levels in the human brain, Pacheco and colleagues recently demonstrated that 9 of 60 healthy < 1-year-old newborns infected with Zika postnatally, later developed neurological complications by 2.5 years of age [23]. Thus, determination here of integrin-αV/β5 levels across a wide range of pediatric ages, and in multiple brain regions, may further define periods during which the CNS remains susceptible to Zika-mediated damage.

GDF15 is a 35-kDa protein, which is cleaved to a 25-kDa product and secreted into the blood [24]. Campderrós and colleagues recently confirmed at the protein level that cold-stress increases the production/secretion of GDF15 in brown adipose tissue (BAT) in mice [25, 26]. GDF15 is produced by numerous other tissues as well, including the choroid plexus epithelium in the cerebral ventricles [27]. Four independent groups in 2017 simultaneously discovered that the orphan receptor GDNF family receptor α-like (GFRAL) mediates GDF15 signaling [28–31]. Specifically, GDF15 bound to GFRAL, complexes with the tyrosine kinase receptor REarranged during Transfection (RET), and together activates downstream intracellular signaling pathways in neurons. Intriguingly, GFRAL receptors are currently thought to be expressed exclusively in the area postrema (AP), and nucleus tractus solitarius (NTS) of the adult hindbrain [31]. The presumed restriction of GFRAL to the AP/NTS is puzzling given that (a) GDF15 knockout (KO) mice develop progressive loss of motor and sensory neurons [32] and (b) direct application of GDF15 to dopaminergic neurons is neuroprotective in vitro and in vivo [33]. Thus, GDF15 promotes neuronal survival in areas outside the AP/NTS but the mechanism(s) responsible are unclear. One possibility is that GFRAL is expressed in regions outside the AP/NTS but like β-klotho follows an age-dependent trajectory that obfuscates its detection [2, 3]. Consistent with that notion, the seminal report on the discovery of GFRAL found that its messenger RNA (mRNA) levels were abundant in the cortex and hippocampus in postnatal mice but absent-to-low in the adult cortex/hippocampus [34]. Thus, we hypothesized that GFRAL is detectable at the protein level in the human infant cortex/hippocampus but absent in adults in those brain regions.

## Experimental Procedures

### Chemicals and Reagents

***mdi Membrane***. We previously demonstrated that polyvinylidene fluoride or polyvinylidene difluoride (PVDF) membranes robustly alter the fidelity of antibodies to detect cold-shock proteins (CSPs) by Western blot analysis [35]. Furthermore, we showed that the mdi brand of membrane has optimal detection properties among the currently available options, germane to the investigation of CSPs/CSHs [3]. All targets were analyzed using 0.2-μM-pore-size mdi membrane, Cat# SVFX8301XXXX101, Lot# VA760606L (mdi Membrane Technologies; Harrisburgh, PA, USA). ***Antibodies***. Table 1 shows the list of primary antibodies used in Western blot studies and vendor-specific details (i.e., host, clonality, catalog #, and lot #). The secondary antibody was a polyclonal anti-rabbit IgG (H+L) cross-absorbed antibody from Fisher (Cat# G-21234; ThermoFisher Scientific; Waltham, MA, USA).

**Table 1:** Primary antibody reagent details. High-specificity monoclonal antibodies were used for the detection of CSH receptor components and MBP. In addition, a higher sensitivity polyclonal antibody targeting GFRAL was also employed

# Target	Vendor	Host	Clonality	Cat #	Lot #
1 MBP	Cell Signaling Technology	Rabbit	Monoclonal	78896	2
2 Integrin *aV*	Cell Signaling Technology	Rabbit	Monoclonal	60896	1
3 Integrin β5	Cell Signaling Technology	Rabbit	Monoclonal	3629	1
4 GFRAL	R&D System	Rabbit	Monoclonal	MAB9697	CMAT 0219111
5 GFRAL	Cusabio	Rabbit	Polyclonal	CSB-PA751020 LA01HU	F0927A

### Human Tissues

Human brain tissues were generously provided by The University of Maryland Brain and Tissue Bank (UMD-BTB) via the NIH NeuroBioBank (NBB) network. Human tissue collections from deceased donors were performed by the UMD-BTB upon receiving informed consent from the donor prior to death, or from the nearest of kin. Human tissue collections by the UMD-BTB is regulated and approved by both the human ethics committees of the Internal Review Board (IRB) of the University of Maryland School of Medicine (study no. HM-HP-00042077) and the IRB of the Department of Health and Mental Hygiene of the State of Maryland. The use of deidentified human samples for research purposes at the University of South Florida was approved by the UMB-BTB after completion of a Material Transfer Agreement (MTA) with the NBB (NBB ID: 782). A detailed description of the rigorous methodology used to prepare protein extracts from these tissues was reported by our group [2]. In addition, we previously described the sample/subject attributes observed in each age-group [2]. In brief, brain tissues from 20 male and 20 female subjects were obtained from the NBB. Hippocampal and BA10 tissues were available for all subjects except for two males in the 3–5-year-old group. For these two males, one had hippocampus available, and the other had BA10 available. Thus, a total of 78 human tissues were analyzed for protein levels of CSH receptors. An equal number of male (*n* = 4) and female (*n* = 4) samples were pooled for analysis into each age cohort, except for the 3–5-year olds which comprised 3 male and 4 female specimens due to sample availability. Thus, groups included infants < 1 year old (*n* = 8, mean age 0.19 + 0.09 years), toddlers aged 1–2 years (*n* = 8, mean age 1.47 + 0.45), early childhood aged 3–5 years (*n* = 7, mean age 4.42 + 0.93), 18-year-old early adolescents (*n* = 8, mean age 18.58 + 0.29), and adults aged 31–34 years (*n* = 8, 33.23 + 1.40). The terminology of each age-range at different neurodevelopmental stages is based on the Eunice Kennedy Shriver National Institute of Child Health and Human Development (NICHD) Pediatric Classification [36]. Samples were homogenized in ice-cold radioimmunoprecipitation assay (RIPA) buffer using a Precellys 24 (Bertin Instruments; Rockville, MD, USA) to generate equivalent homogenates. Protein concentrations were adjusted to ~ 3.5 μg/μL. Deidentified NBB subject codes are indicated in the supplementary figures and searchable via the NBB portal.

### Western Blot

Protein analysis was done as described by our group and employed to measure the relative levels of components of CSH receptors in five human age-cohorts [2]. Chemiluminescence was measured on a 9.1MP CL1500 iBright (ThermoFisher Scientific). Binning was set to 5 × 5 to increase signal-to-noise for band detection. Images were exported as 600 dpi tagged image file format (TIFF) files. All blots corresponding to graphs in primary figures show unaltered original images (i.e., blots and total protein stains were not contrast/brightness modified). An enhanced image (i.e., brightness/contrast adjusted) is included in the supplemental data for cortical integrin-β5 blots; the enhanced images were not used for densitometry but to aid visualization of signals. Individual blots were performed for each target across a given sample set. Thus, for each target per brain region, 39 samples were divided equally (by age and gender) across 2× 26-well Criterion gels (BioRad; Hercules, CA, USA). Densitometry of proteins and total stain were obtained using UN-SCAN-IT software (Silk Scientific; Orem, UT, USA). To correct for loading/transfer errors (i.e., to standardize), target protein densitometry was divided by the densitometry of the total protein stain corresponding to each lane within the respective membrane. Standardized densitometric values for each blot were normalized by dividing by the largest intra-blot value (i.e., the highest value in each blot was set to 100%). Data are expressed as the relative differences in target expression compared by age groups. Normalized values (across all 39 samples) were pooled for statistical analysis. Figures were compiled in Photoshop (Adobe; San José, CA, USA).

### Liquid Chromatography with Tandem Mass Spectrometry

Mass spec was employed to determine if GFRAL protein spectra could be detected in infant vs. adult brain samples. In brief, samples were electrophoresed on a 4–20% mini-protean TGX precast gel and bands visualized with Coomassie stain. Gel segments spanning the 30–50 kDa range were extracted and de-stained (i.e., flanking the faint ~ 44 kDa presumptive GFRAL signal seen in infants). Gel slices were washed, subjected to in-gel reduction with 2-mM Thermo Scientific Bond-Breaker (TCEP) in ammonium bicarbonate (AmBic), and alkylated with 20-mM iodoacetamide in AmBic to prevent disulfide bond reformation. Gel slices were trypsinized (20 ng/μL) in AmBic. Peptide extraction was performed in 100 μL 50% acetonitrile/0.1% trifluoroacetic acid (TFA) and proteins characterized on a Thermo Q-exactive-HFX mass spectrometer coupled to a Thermo Easy nLC 1200 (ThermoFisher Scientific). The mass spectrometer was outfitted with a Thermo Nanospray Flex source with the following parameters: spray voltage 2.24, capillary temperature 200 °C, funnel RF level = 40. Parameters for data acquisition were as follows: for mass spec (MS) data, the resolution was 60,000 with an automatic gain control (AGC) target of 3e6 and a max IT time of 50 ms; the range was set to 400–1600 m/z. MS/MS data was acquired with a resolution of 15,000, an AGC of 1e5, max IT of 50 ms, and the top 30 peaks were picked with an isolation window of 1.6 m/z with a dynamic execution of 25 s. Samples were searched using Thermo Proteome Discoverer v2.2.0.388. Groups were assigned as infants (A) vs. adults (B) and searched using the default label-free quant processing and consensus programs against a Uniprot Human database.

### Statistics

Normalized Western blot densitometry values, expressed as the relative difference in target levels, were analyzed by nonparametric tests. The effect of developmental age on target expression was analyzed using the Kruskal-Wallis 1-way analysis of variance (ANOVA) on ranks test followed by Dunn’s post hoc multiple-comparisons test. Gender was analyzed by the Mann-Whitney *U* test. Data were analyzed using GraphPad Prism software (GraphPad Inc., La Jolla, CA, USA). Data are significant at *p* < 0.05. MS were analyzed in Proteome Discoverer. The results of identified gene targets are categorized by false-discovery rate (FDR)-Confidence ratings (high, 1%; medium, 5%; low, 10%) and *p* values on the abundance ratio of (A)/(B) are indicated (Supplementary Table 1).

## Results and Discussion

Robust and rigorous studies on baseline CSP/CSH levels in the human brain will help to inform on these important proteins for fundamental studies in neurobiology and neuroscience, and could also aid in defining the patient populations most likely to benefit from future therapies aimed at augmenting individual components of the molecular cold-stress response, as a novel approach to enhancing the neuroprotective efficacy of targeted temperature management in the neonatology and neurocritical care [1]. We previously obtained 78 high-quality male/female human brain tissue specimens from the NBB, which encompassed five cohorts (infant, toddler, preschooler, adolescent, and adult), to investigate the neurodevelopmental time course of three CSPs and β-klotho in hippocampal and Brodmann area 10 (BA10) cortex [2]. Here, we used these protein extracts to measure the levels of GFRAL, integrin-αV, and integrin-β5.

**Fig. 1:**
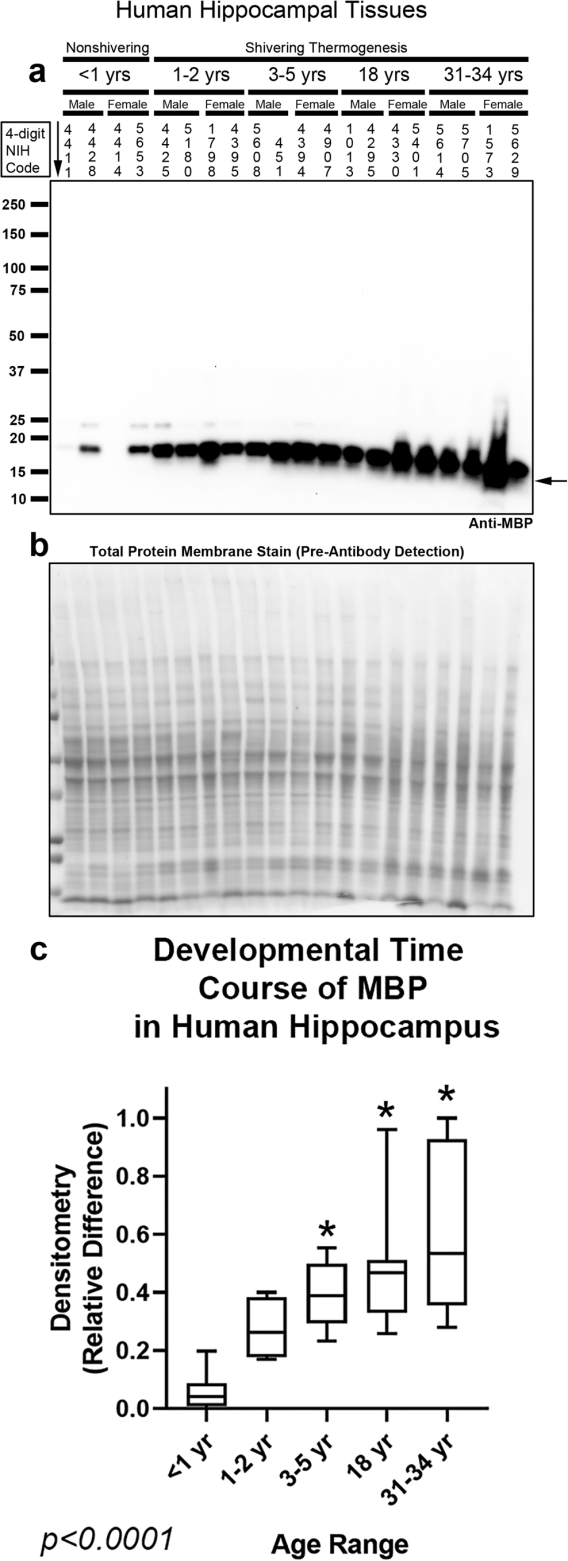
Developmental time course of myelin basic protein (MBP) in the Human Hippocampus. **a** Representative blot of relative MBP levels in the hippocampus in infants (*n* = 8/group), toddlers (*n* = 8/group), preschoolers(*n* = 7/group), adolescents (*n* = 8/group), and adults (*n* = 8/group). **b** Membrane stain shows total protein loading across subjects. **c** Normalized densitometry values were analyzed by Kruskal-Wallis 1-way ANOVA. * indicates post hoc significance vs. infants using the Dunn’s multiple comparison test. Data were significant at *p* < 0.05. Box plots show minimum, maximum, IQR, and median. The age-appropriate mechanism of thermogenesis used in humans (non-shivering vs. shivering) is denoted above age-cohorts

**Fig. 2:**
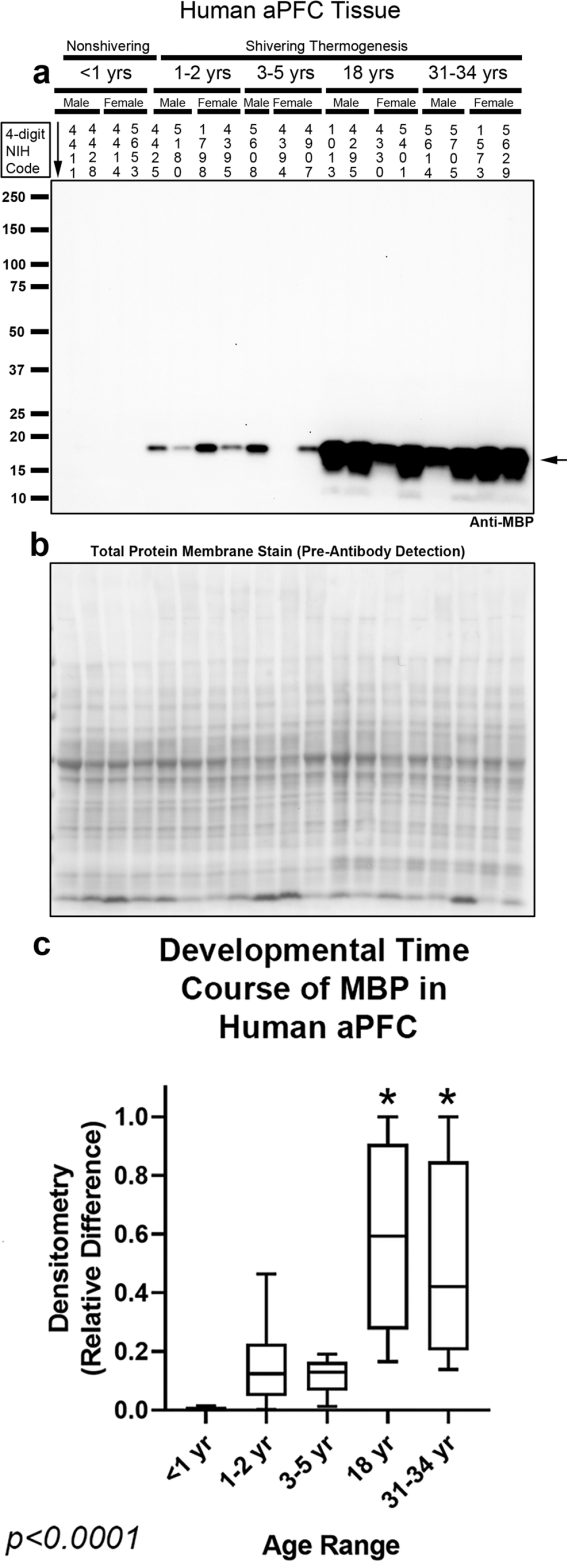
Developmental time course of myelin basic protein (MBP) in the human anterior prefrontal cortex (aPFC). **a** Representative blot of relative MBP levels in aPFC in infants (*n* = 8/group), toddlers (*n* = 8/group), preschoolers (*n* = 7/group), adolescents (*n* = 8/group), and adults (*n* = 8/group). **b** Membrane stain shows total protein loading across subjects. **c** Normalized densitometry values were analyzed by Kruskal-Wallis 1-way ANOVA. * indicates post hoc significance vs. infants using the Dunn’s multiple comparison test. Data were significant at *p* < 0.05. Box plots show minimum, maximum, IQR, and median

In prior studies, we observed that CSPs were consistently robust in infants, followed by a precipitous decline with advancing age. To substantiate that instances in which CSPs/CSH receptors are only found in infants were not caused by extraneous factors like differences in sample quality, we measured myelin basic protein (MBP). Detection of MBP with a high-specificity rabbit monoclonal antibody revealed that levels progressively increased with age in the hippocampus (Fig. 1 and Supplementary Fig. 1) and cortex (Fig. 2 and Supplementary Fig. 2), which supported the integrity of our samples.

**Fig. 3:**
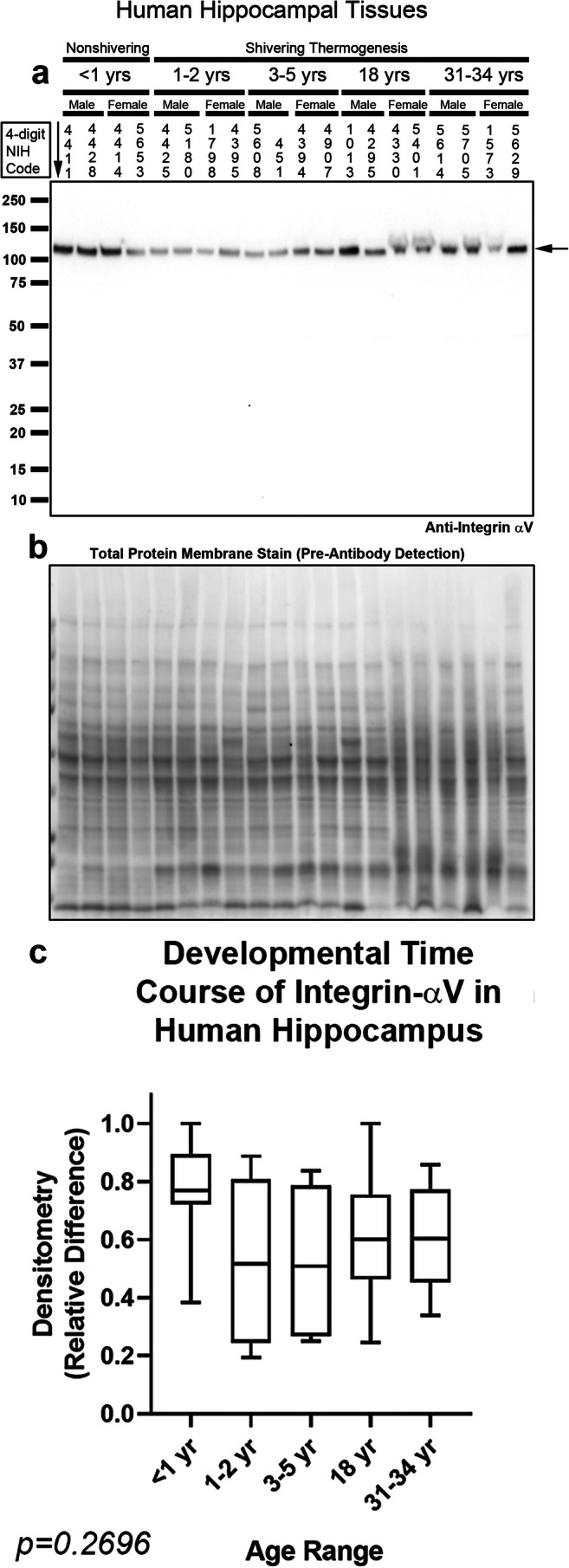
Developmental time course of integrin-αV in the human hippocampus. **a** Representative blot of relative integrin-αV levels in the hippocampus in infants (*n* = 8/group), toddlers (*n* = 8/group), preschoolers (*n* = 7/group), adolescents (*n* = 8/group), and adults (*n* = 8/group). **b** Membrane stain shows total protein loading across subjects. **c** Normalized densitometry values were analyzed by Kruskal-Wallis 1-way ANOVA. Data were significant at *p* < 0.05. Box plots show minimum, maximum, IQR, and median

**Fig. 4:**
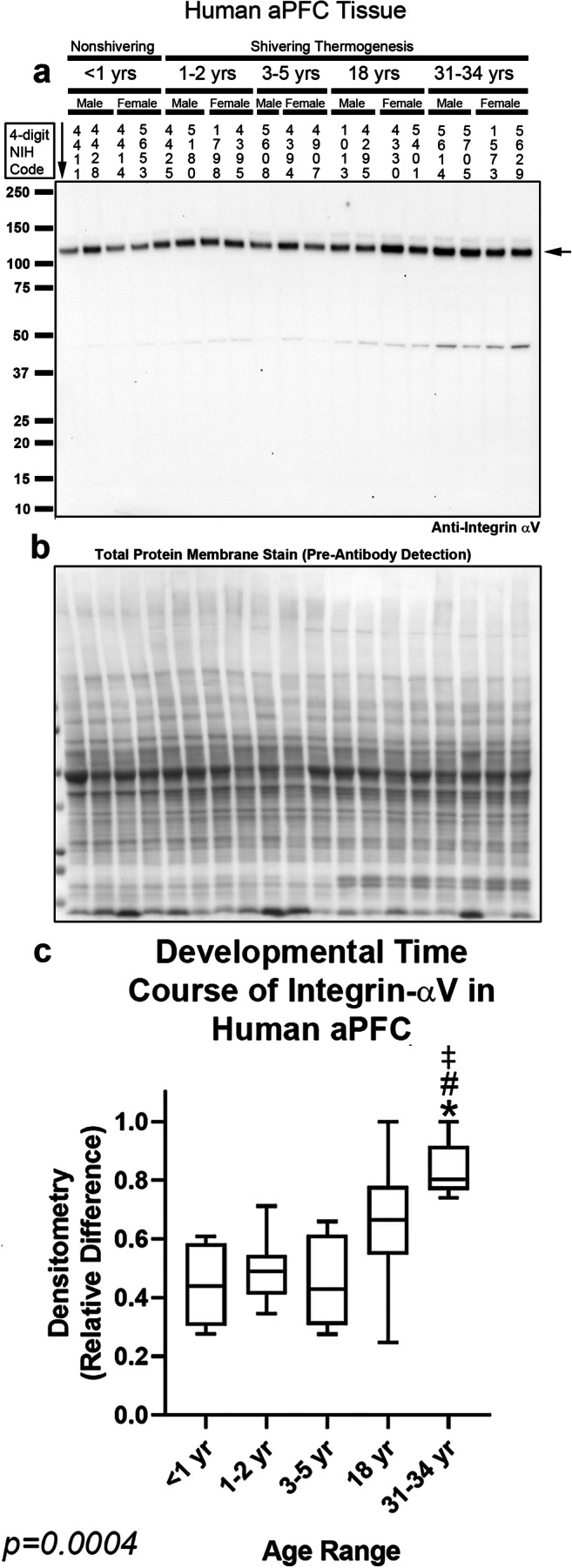
Developmental time course of integrin-αV in the human anterior prefrontal cortex (aPFC). **a** Representative blot of relative integrin-αV levels in aPFC in infants (*n* = 8/group), toddlers (*n* = 8/group), preschoolers (*n* = 7/group), adolescents (*n* = 8/group), and adults (*n* = 8/group). **b** Membrane stain shows total protein loading across subjects. **c** Normalized densitometry values were analyzed by Kruskal-Wallis 1-way ANOVA. * indicates post hoc significance vs. infants. # indicates post hoc significance vs. toddlers. ‡ indicates post hoc significance vs. preschoolers, using the Dunn’s multiple comparison test. Data were significant at *p* < 0.05. Box plots show minimum, maximum, IQR, and median

**Fig. 5:**
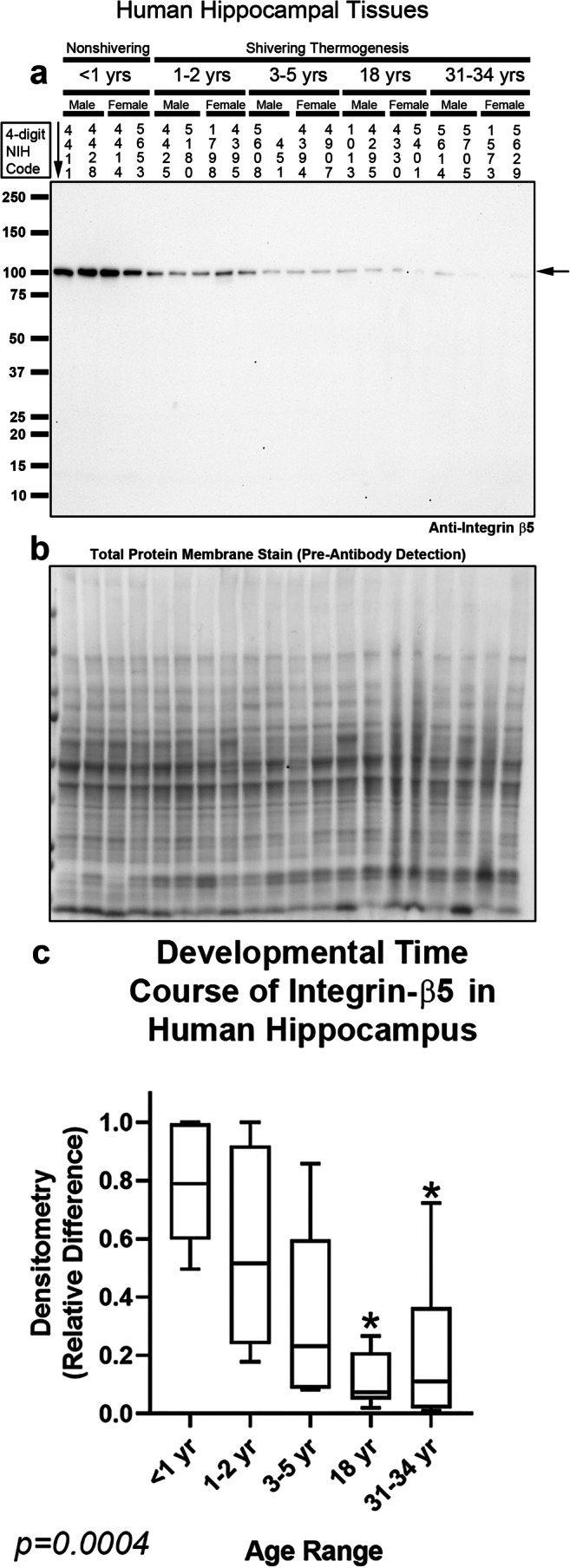
Developmental time course of integrin-β5 in the human hippocampus. **a** Representative blot of relative integrin-β5 levels in the hippocampus in infants (*n* = 8/group), toddlers (*n* = 8/group), preschoolers (*n* = 7/group), adolescents (*n* = 8/group), and adults (*n* = 8/group). **b** Membrane stain shows total protein loading across subjects. **c** Normalized densitometry values were analyzed by Kruskal-Wallis 1-way ANOVA. * indicates post hoc significance vs. infants using the Dunn’s multiple comparison test. Data were significant at *p* < 0.05. Box plots show minimum, maximum, IQR, and median

**Fig. 6:**
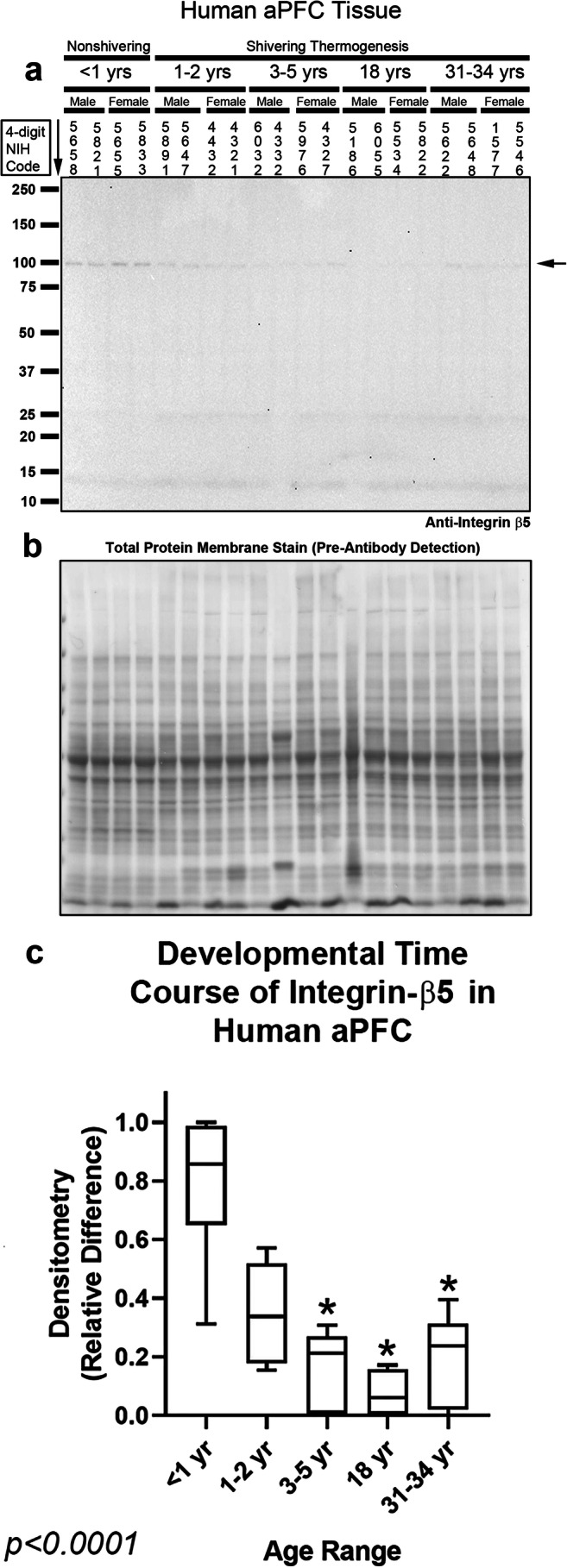
Developmental time course of integrin-β5 in the human anterior prefrontal cortex (aPFC). **a** Representative blot of relative integrin-β5 levels in aPFC in infants (*n* = 8/group), toddlers (*n* = 8/group), preschoolers (*n* = 7/group), adolescents (*n* = 8/group), and adults (*n* = 8/group). **b** Membrane stain shows total protein loading across subjects. **c** Normalized densitometry values were analyzed by Kruskal-Wallis 1-way ANOVA. * indicates post hoc significance vs. infants using the Dunn’s multiple comparison test. Data were significant at *p* < 0.05. Box plots show minimum, maximum, IQR, and median

Integrin receptors comprise a diverse family of integrin-α and β subunits. There are at least 21 different integrin receptors in the brain, based on permutations in their subunit dimerization [37]. Integrin-αV/β5 heterodimers were recently identified as the primary receptor unit that mediates intracellular signaling responses to the CSH irisin [14]. Thus, we measured integrin-αV and integrin-β5 levels with high-specificity rabbit monoclonal antibodies. Integrin-αV migrates at ~ 130 kDa on sodium dodecyl sulfate polyacrylamide gel electrophoresis (SDS-PAGE) under reducing conditions [38]. Integrin-αV was detected in the hippocampus in all subjects and unaffected by age (Fig. 3 and Supplementary Fig. 3). Integrin-αV was also detected at all ages in the prefrontal cortex but levels were significantly increased in adults vs. infants, toddlers, and preschoolers (Fig. 4 and Supplementary Fig. 4). Integrin-β5 migrates at ~ 100 kDa on SDS-PAGE under reducing conditions [39]. Contrasting the findings with integrin-αV, in the hippocampus, integrin-β5 was significantly increased in infants vs. adolescent and adults (Fig. 5 and Supplementary Fig. 5). In the prefrontal cortex, integrin-β5 was detected mainly in infants (Fig. 6, Supplementary Fig. 6, and Supplementary Fig. 7).

Co-expression of integrin-αV/β5 in the human infant hippocampus and cortex suggests the possibility that irisin has a more robust effect on the developing vs. the adult brain. This seems counterintuitive as irisin is primarily secreted by skeletal muscle in response to exercise or shivering, and infants lack the ability for both (i.e., skeletal muscle is underdeveloped in newborns and body heat is primarily maintained via non-shivering thermogenesis in the first year of life) [40]. Thus, we expected that the adult brain would have higher integrin-αV/β5 levels. A few caveats merit mention. First, integrin-αV/β5 receptors are not selective for irisin but also bind secreted glycoprotein milk-fat globule factor-E8 (MFG-E8) and the extracellular matrix protein vitronectin [41, 42]. Thus, ligands other than irisin could play a dominant role in stimulating integrin-αV/β5 receptors in the infant brain. Second, the source of irisin in infants is unclear. Irisin levels are robustly increased in mothers vs. infants, which is consistent with adults having higher muscle mass [43]. Also, labor promotes maternal irisin secretion [44]. Thus, while irisin is detectable in cord blood in human newborns, whether it is derived from the mother or endogenously produced remains to be determined [45]. Nevertheless, the abundance of receptor components in the infant hippocampus and cortex raise the possibility that exogenous supplementation with irisin may have benefits on the newborn brain and potentially in the setting of therapeutic hypothermia.

Integrin-αV/β5 receptors also mediate Zika virus infection of neural progenitor cells [16, 17]. Moreover, integrin-β5 represents the internalization factor that mediates neurotropism [16]. Zika virus causes devastating deformations (microcephaly) to the developing brain but the risk of CNS damage is considered greatest during pregnancy (i.e., injury to the fetal brain in utero) [46, 47]. However, a 2020 report called into question the timing of the “susceptibility window” by demonstrating that inoculating 5-week-old infant macaques with Zika (equivalent to a 4-month-old human infant) resulted in enlarged ventricles, damage to multiple brain structures, and produced a variety of behavioral deficits [48]. Consistent with those findings, Pacheco and colleagues showed that 15% of patients, in a cohort of < 1-year-old infants postnatally infected with Zika, later developed long-term neurological abnormalities. Prior analyses on integrin-β5 expression in the human brain were limited to adults; levels were abundant in resected CNS gliomas but absent in normal/healthy brain tissue (consistent with findings here) [49]. To our knowledge, our report is the most robust analysis to date on neurodevelopmental changes in integrin-αV/β5 protein levels in the human brain. Our results on integrin-β5 levels in infants raise important clinical questions and further support prior studies indicating that the window of susceptibility for Zika-induced neural damage in children extends beyond the 3rd trimester. Also, we found that integrin-β5 was readily detectable in the hippocampus in infants and in 1–2-year-old toddlers but primarily in infants in the prefrontal cortex. Given that experimentally blocking integrin-αV/β5 receptors attenuated Zika-induced neuropathology in mice—our findings offer new clues germane to the postnatal ages and brain regions in humans during which integrin-αV/β5 targeting therapies may be most effective [16].

**Fig. 7:**
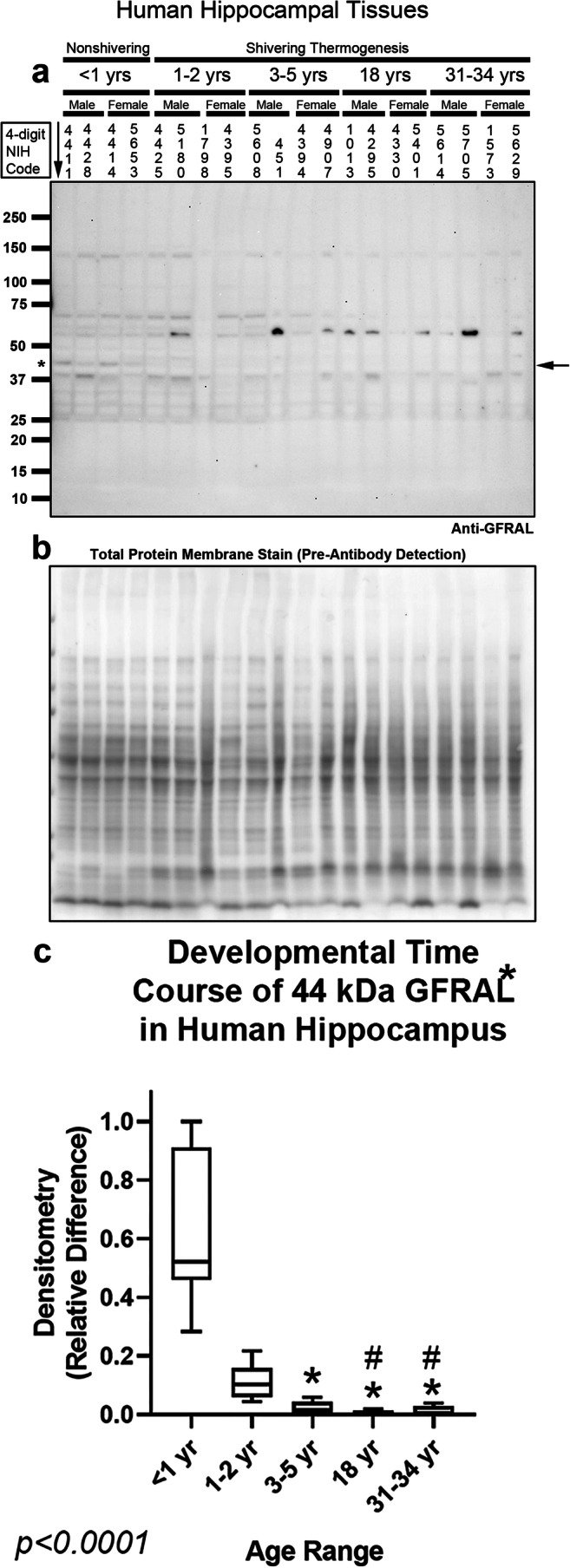
Developmental time course of GFRAL in the human hippocampus. **a** Representative blot of relative GFRAL levels in the hippocampus in infants (*n* = 8/group), toddlers (*n* = 8/group), preschoolers (*n* = 7/group), adolescents (*n* = 8/group), and adults (*n* = 8/group). **b** Membrane stain shows total protein loading across subjects. **c** Normalized densitometry values were analyzed by Kruskal-Wallis 1-way ANOVA. * indicates post hoc significance vs. infants. # indicates post hoc significance vs. toddlers. Data were significant at *p* < 0.05. Box plots show minimum, maximum, IQR, and median. The asterisk above GFRAL denotes that the band analyzed by densitometry matches the predicted kDa

**Fig. 8:**
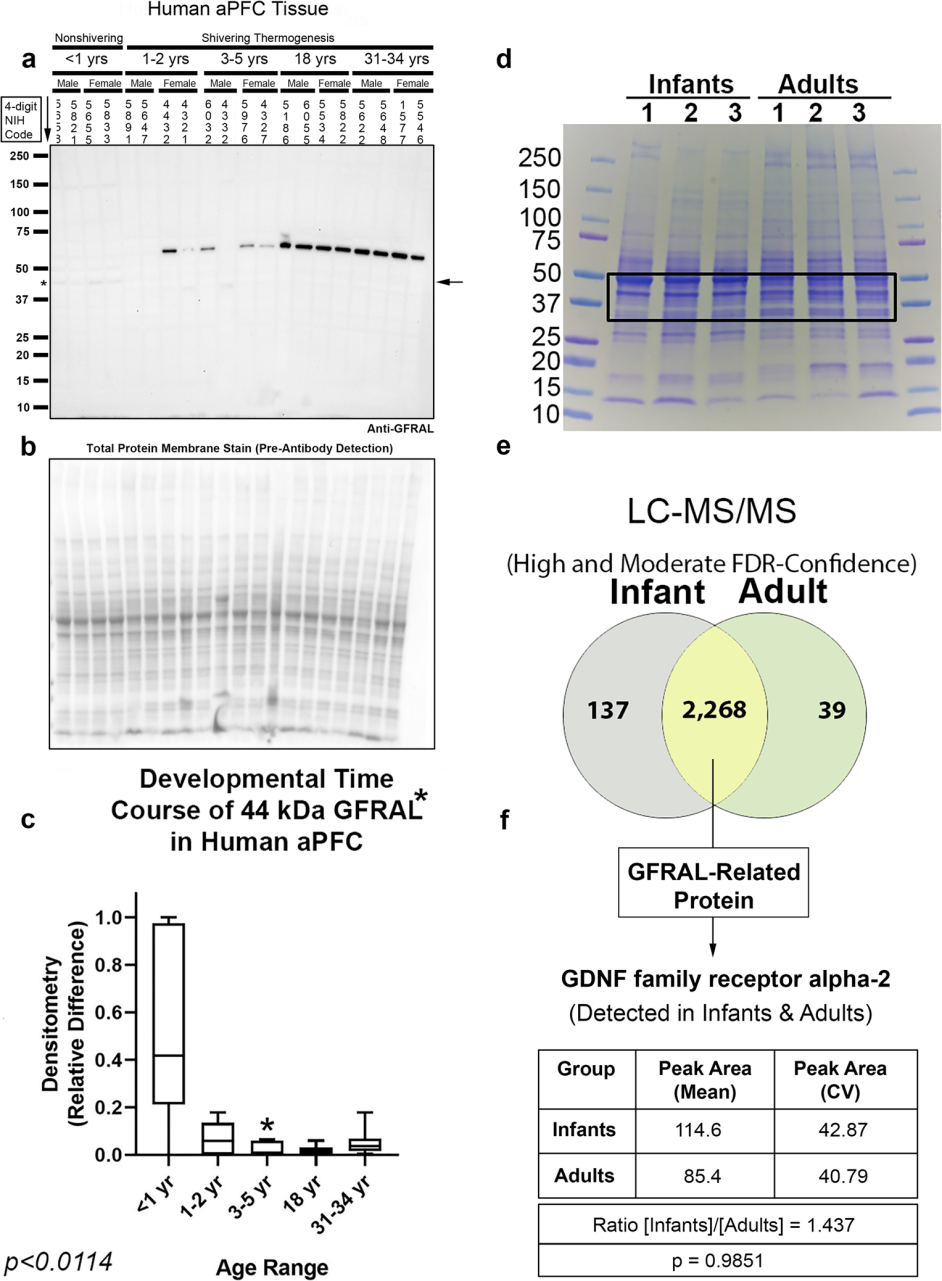
Developmental time course of GFRAL in the human anterior prefrontal cortex (aPFC). **a** Representative blot of relative GFRAL levels in aPFC in infants (*n* = 8/group), toddlers (*n* = 8/group), preschoolers (*n* = 7/group), adolescents (*n* = 8/group), and adults (*n* = 8/group). **b** Membrane stain shows total protein loading across subjects. **c** Normalized densitometry values were analyzed by Kruskal-Wallis 1-way ANOVA. * indicates post hoc significance vs. infants. **d** MS analysis of cortical proteins spanning the 30–50-kDa range in infants (*n* = 3) vs. adults (*n* = 3). **e** Venn diagram summarizes the results of Proteome Discoverer analysis and the number of unique genes detected only in infants (137) vs. adults (39) for high and medium FDR-confidence targets. **f** GFR2α, a protein related to GFRAL, was detected in all groups but did not significantly differ in infants vs. adults (*p* = 0.9851). Data were significant at *p* < 0.05. Box plots show minimum, maximum, IQR, and median. The asterisk above GFRAL denotes that the band analyzed by densitometry matches the predicted kDa

GFRAL was recently identified as the obligatory receptor for GDF15, and is predicted to migrate on SDS-PAGE at ~ 44 kDa [29]. We identified a single commercially available high-specificity monoclonal anti-GFRAL antibody from R&D Systems, which was recommended for enzyme-linked immunosorbent assay (ELISA) and flow-cytometry applications but did not specify use in Western blot experiments. The monoclonal antibody failed to detect a signal across the age-spectrum in human hippocampal homogenates (Supplementary Fig. 8a). Next, we tested a polyclonal anti-GFRAL antibody from Cusabio on the same blot—to take advantage of the increased sensitivity afforded by polyclonals—which produced a broad spectrum of bands across the molecular weight range including a faint ~ 44 KDa band which was only seen in infants (Fig. 7, and Supplementary Fig. 8b). In the prefrontal cortex, a fewer number of bands were detected with the Cusabio antibody (Fig. 8a, and Supplementary Fig. 9). Notably, the faint ~ 44 kDa band was again observed only in infants and not seen in older age groups. In attempt to further validate the identity of the faint ~ 44 kDa band detected by the polyclonal antibody, cortical homogenates from infants and adults (ID 4411, 5655, 5833, 5622,1573, and 5629) were selected for mass spectrometry to analyze the presence of GFRAL signals in the 30–50-kDa range (Fig. 8d). GFRAL spectra were not included among the 2268 protein targets detected in both infants and adults, but we were able to detect 137 unique protein targets, expressed only in infants (Fig. 8e, and Supplementary Table 1). However, mass spectrometry did identify the related GFRAL gene/protein GDNF family receptor alpha-2 (GFR2α) (Fig. 8f and Supplementary Table 1). GFR2α abundance was not significantly different in infants vs. adults (Fig. 8f), suggesting that the faint ~ 44 kDA band seen in infants is a different/unrelated protein. Finally, there were no significant sex differences in the levels of targets analyzed by Western blot (Supplementary Fig. 10).

Reports on the distribution of GFRAL mRNA expression in the brain are contradictory. Li and colleagues first cloned GFRAL and used polymerase chain reaction (PCR) analysis to show that mRNA levels were abundant in total mouse brain extracts but absent in the heart, spleen, lung, liver, kidney, placenta, skeletal muscle, and small intestine [34]. Furthermore, within the brain, GFRAL mRNA was abundant in the postnatal pup cortex and hippocampus but low-to-absent in these regions in adults. Finally, GFRAL mRNA was also detected at various levels in the adult substantia nigra, septum, thalamus, and in the spinal cord [34]. In contrast, Hsu and colleagues reported that GFRAL mRNA in the mouse brain was present only in the AP/NTS, and absent in the cortex, hippocampus, midbrain, and spinal cord [31]. Moreover, Mullican and colleagues reported that brain GFRAL mRNA expression levels in humans are different than in mice, and more widespread in the former (albeit at low levels) [29]. Thus, we hypothesized that GFRAL protein levels might be increased in regions outside the AP/NTS at an early age. Measurement with a high-specificity (lower sensitivity) monoclonal anti-GFRAL antibody or mass spectrometry failed to detect GFRAL in the human prefrontal cortex. Given limitations in the sensitivity of label-free LC-MS/MS to detect low-abundant proteins in complex samples, we cannot rule out the possibility that the very faint ~ 44 kDa band in infants detected with a polyclonal antibody is authentic GFRAL expressed at very low levels. Regardless, our findings further support work by others indicating that GFRAL protein is not expressed at biologically meaningful levels in the hippocampus/cortex across a wide range of neurodevelopmental stages in humans.

## Conclusion

In summary, here, we analyzed CSH receptor levels in the human brain utilizing a large/diverse high-quality cohort of 78 brain tissue samples, with equal representation of sex as a variable, and spanning five distinct neurodevelopmental stages. Integrin-αV was present in all ages in both the cortex and hippocampus. Integrin-αV levels increased with age in the cortex. Integrin-β5 was primarily expressed in infants in the cortex, and in a range of younger subjects in the hippocampus (infants, toddlers, and preschoolers), but not adults. The neurodevelopmental expression pattern of integrin-β5 was similar to our prior findings on CSPs (RNA Binding Motif Protein 3, RBM3; Cold Inducible RNA Binding Protein, CIRBP, Reticulon 3, RTN3) and β-klotho in these samples. The mounting evidence that numerous CSP/CSH proteins are uniquely enriched in the infant human brain is further supported by the robust age-dependent increase in MBP levels (positive control) which further validates the quality of our sample set. In contrast, GFRAL levels were not robustly increased in either the cortex or the hippocampus in any of the age groups, including infants or early childhood, refuting our hypothesis. Our findings support the need for additional research on irisin particularly in the developing brain. Future studies are needed to confirm that irisin activates integrin-αV/β5 receptors in neonates and to confirm a role in neuroprotection. Finally, our findings could have important additional implications given the roles of integrin-αV/β5 receptors in Zika virus infection.

## Supplementary Information

ESM 1 (DOCX 2100 kb)

ESM 2 (XLSX 558 kb)

## Data Availability

All data germane to this work is provided in the primary and electronic supplementary material. Human brain tissues can be obtained by submitting a formal request to the NBB (https://neurobiobank.nih.gov/).
