# Supplementary material for: Hippocampal and Prefrontal Cortical Brain Tissue Levels of Irisin and GDF15 Receptor Subunits in Children
Source: Mol Neurobiol. 2021 Jan 7;58(5):2145–57. doi: 10.1007/s12035-020-02250-4 (PMC7788542; doi:10.1007/s12035-020-02250-4)
Supplement: Supplementary file 1 — (DOCX 2100 kb) [file 12035_2020_2250_MOESM1_ESM.docx]

**Title:** **Hippocampal and Prefrontal Cortical Brain Tissue Levels of Irisin and GDF15 Receptor Subunits in Children.**

**Running Title:** CSH Receptors in the Human Brain.

**Authors:** Travis C. Jackson,^1,2*^ Kiersten Gorse^1,2^, Jeremy R. Herrmann^3,4^, and Patrick M. Kochanek^3,4^

**Affiliations:**

^1^University of South Florida

Morsani College of Medicine

USF Health Heart Institute, MDD 0742

560 Channelside Dr

Tampa FL, 33602

USA

^2^Department of Molecular Pharmacology & Physiology

University of South Florida, Morsani College of Medicine

12901 Bruce B Downs BLDV, MDC 2532

Tampa FL, 33612-4799

USA

^3^Safar Center for Resuscitation Research

University of Pittsburgh, School of Medicine

Children’s Hospital of Pittsburgh of UPMC

John G. Rangos Research Center – 6th Floor

4401 Penn Avenue

Pittsburgh, PA 15224

USA

^4^Department of Critical Care Medicine

University of Pittsburgh, School of Medicine

Scaife Hall

3550 Terrace Street

Pittsburgh, PA 15213

USA

*Corresponding Author

University of South Florida

Morsani College of Medicine

USF Health Heart Institute, MDD 0742

560 Channelside Dr

Tampa FL, 33602

Email: [tcjackson@usf.edu](mailto:tcjackson@usf.edu)

ORCiD: **0000-0003-2749-7881**

**Figures**

Fig. S1





Fig. S2





Fig. S3





Fig. S4





Fig. S5





Fig. S6





Fig. S7





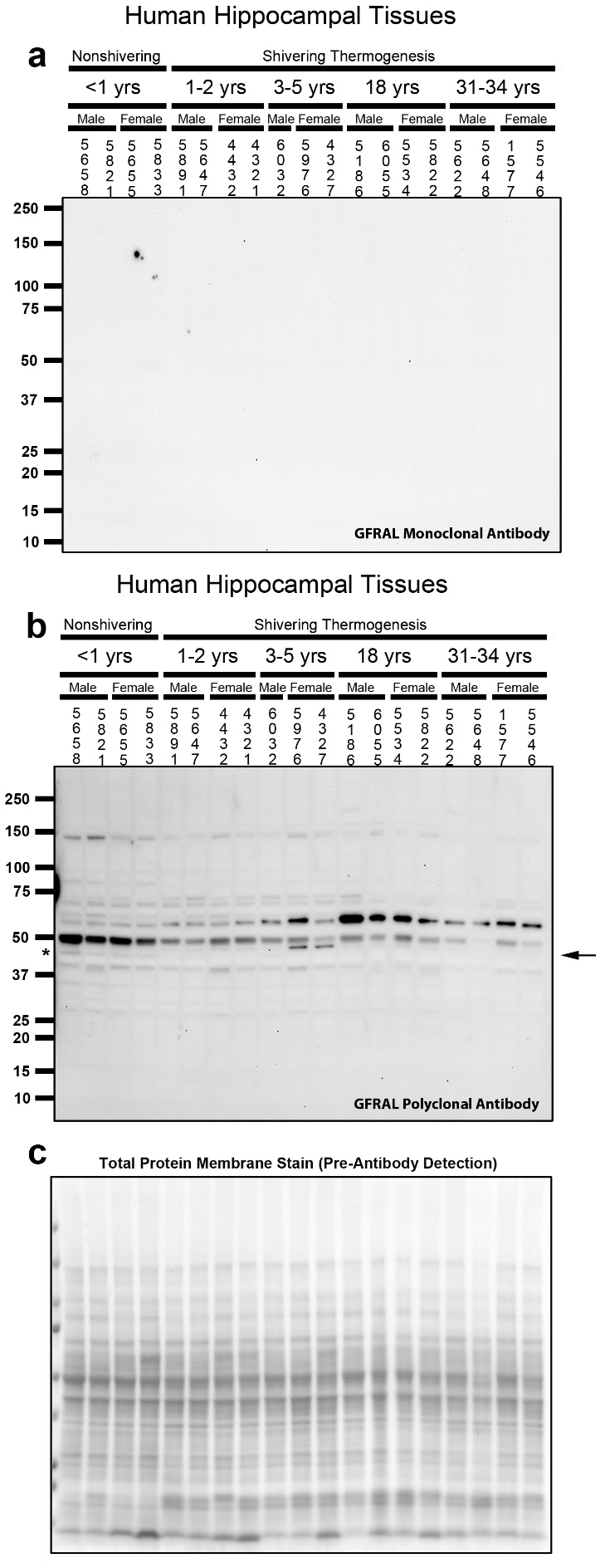
Fig. S8

Fig. S9


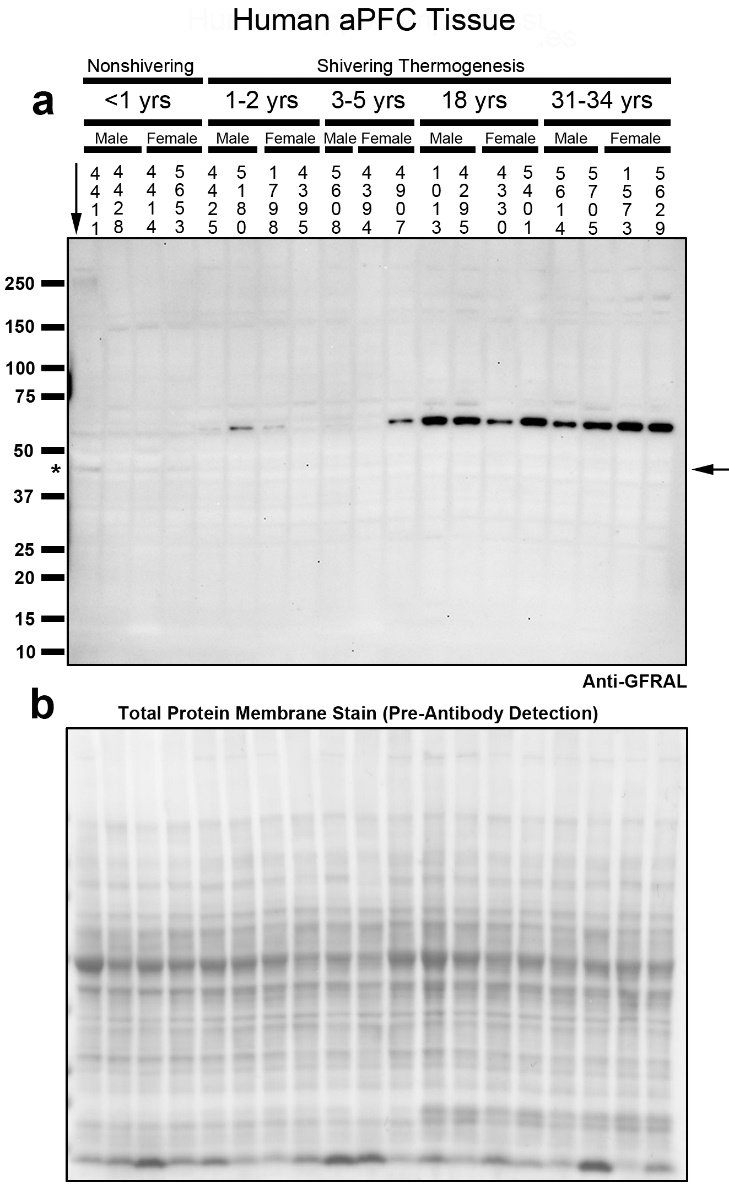


Fig. S10
